# pcMSC Modulates Immune Dysregulation in Patients With COVID-19-Induced Refractory Acute Lung Injury

**DOI:** 10.3389/fimmu.2022.871828

**Published:** 2022-04-29

**Authors:** Mei-Chuan Chen, Kevin Shu-Leung Lai, Ko-Ling Chien, Sing Teck Teng, Yuh-Rong Lin, Wei Chao, Meng-Jung Lee, Po-Li Wei, Yen-Hua Huang, Han-Pin Kuo, Chih-Ming Weng, Chun-Liang Chou

**Affiliations:** ^1^ Division of Pulmonary Medicine, Department of Internal Medicine, Taipei Medical University Hospital, Taipei, Taiwan; ^2^ Pulmonary Medicine Research Center, Taipei Medical University, Taipei, Taiwan; ^3^ Department of Critical Care Medicine, Taipei Medical University Hospital, Taipei, Taiwan; ^4^ Division of Colorectal Surgery, Department of Surgery, Taipei Medical University Hospital, Taipei, Taiwan; ^5^ Department of Surgery, College of Medicine, Taipei Medical University, Taipei, Taiwan; ^6^ Graduate Institute of Cancer Biology and Drug Discovery, Taipei Medical University, Taipei, Taiwan; ^7^ Department of Biochemistry and Molecular Cell Biology, School of Medicine, College of Medicine, Taipei Medical University, Taipei, Taiwan; ^8^ School of Respiratory therapy, College of Medicine, Taipei Medical University, Taipei, Taiwan

**Keywords:** COVID-19, mesenchymal stem cell, severe lung injury, immune response, Treg cells

## Abstract

**Background and Objectives:**

The novel coronavirus disease 2019 (COVID-19) has been a pandemic health issue in 30 January 2020. The mortality rate is as high as 50% in critically ill patients. Stem cell therapy is effective for those who are refractory to standard treatments. However, the immune responses that underlie stem cell therapy have not been well reported, particularly, in patients associated with moderate to severe acute respiratory distress syndrome (ARDS).

**Methods:**

On Days 0 and 4, an intravenous infusion of 2 × 10^7^ placenta-derived mesenchymal stem cells (pcMSCs) (MatriPlax) were administered to five severe COVID-19 patients refractory to current standard therapies. Peripheral blood inflammatory markers and immune profiles were determined by multi-parameter flow cytometry and studied at Days 0, 4, and 8. Clinical outcomes were also observed.

**Results:**

None of the pc-MSC treated patients experienced 28-day mortality compared with the control group and showed a significant improvement in the PaO_2_/FiO_2_ ratio, Murray’s lung injury scores, reduction in serum ferritin, lactate dehydrogenase (LDH), and C-reactive protein (CRP) levels. The cytokine profiles also showed a reduction in IL-1β, IFN-γ, IL-2, and IL-6, and an increase in IL-13 and IL-5 type 2 cytokines within 7 days of therapy. Lymphopenia was also significantly improved after 7 days of treatment. Immune cell profiles showed an increase in the proportions of CD4^+^ T cells (namely, CD4^+^ naïve T cells and CD4^+^ memory T cell subtypes), Treg cells, CD19^+^ B cells (namely, CD19^+^ naïve B cells, CD27^+^ switched B cell subtypes) and dendritic cells, and a significant decrease in the proportion of CD14^+^ monocytes (namely, CD16^-^ classical and CD16^+^ non-classical subtypes), and plasma/plasmablast cells. No adverse effects were seen at the serial follow-up visits for 2 months after initial therapy.

**Conclusion:**

pc-MSCs therapy suppressed hyper-inflammatory states of the innate immune response to COVID-19 infection by increasing Treg cells, decreasing monocytes and plasma/plasmablast cells, and promoting CD4^+^ T cells and CD19^+^ B cells toward adaptive immune responses in severely critically ill COVID-19 patients with moderate to severe ARDS, especially those who were refractory to current standard care and immunosuppressive therapies.

## Introduction

The novel coronavirus disease 2019 (COVID-19) has been a pandemic health issue since 30 January 2020 (1). Among those infected across the world, approximately 80% of the cases experienced mild disease. However, nearly 20% of infected patients progress to severe clinical courses, of which 5% are considered critical cases (2). In critically ill patients, mortality rates were superior to 50% (3, 4). The severity of COVID-19 infection varies among individuals (5) and is attributed to differences in the magnitude of the dysregulated cytokine responses to viral infection (6). Immunomodulatory therapies, namely, dexamethasone (7), tocilizumab (8), and barcitinib (9) have been applied to restore immune dysregulation processes and are effective in reducing mortality in some severe COVID-19 patients. However, despite the administration of immunotherapies, COVID-19 could lead to high mortality rates among patients admitted to intensive care units (ICU) with severe respiratory failure (10). To date, many emerging inhibitors of specific inflammatory pathways have been proposed (1). However, most treatments are waiting to be validated in large-scale trials.

Mesenchymal stem cells (MSCs) are multipotent adult stem cells with variable magnitudes of proliferation and activity. They are derived from various tissues and organs, including placenta choriodecidual-membranes (11, 12). The beneficial effects of MSCs in alleviating disease are attributed to their potential to modulate cytokine secretion, inflammatory cell migration, and immune function responses, and more specifically, their immunosuppressive properties against dendritic cells, NK cells, monocytes, macrophages, and T cells. Furthermore, this response is often associated with a concomitant increase in the regulatory T cell (Treg) fraction (13–17).

For treating pulmonary diseases, MSCs present a notable strength due to their ability to be retained in the lungs for a period ranging from hours to days (13). The therapeutic effects of MSC-based therapies in ARDS have been described in several experimental and clinical studies, including COVID-19 (18). A MSC clinical trial in severe COVID-19 patients conducted in the United States demonstrated benefits in attenuating clinical severity and associated cytokine changes (19). The immunomodulatory effects of MSCs in one case of COVID pneumonia with mild ARDS have been previously studied in China (20). Recent studies have also revealed improved clinical outcomes with MSC therapy, with reported reductions in mortality and morbidity rates amongst COVID-19 patients (21–23). However, little is known regarding the specific details of its immune-modulatory effect.

MSCs from placenta choriodecidual membranes (pcMSCs, Matriplax) consist of *ex vivo* culture-expanded human MSCs isolated from the placenta choriodecidual membranes of healthy adult mothers and have a high penetration capacity into lung tissue (12). pcMSCs suppress airway inflammation in asthmatic rats by increasing the number of Treg cells and are accompanied by a reduced presence of Th17 cells, macrophages, neutrophils, and eosinophils that infiltrate the lung. They have also been reported to improve survival and promote recovery in animal models with LPS-induced acute lung injury (ALI) (24, 25). Thus, treatment with pcMSCs is anticipated to be a feasible therapy for patients with severe or critically ill COVID-19. In this study, we evaluated the safety and clinical efficacy of pcMSC therapy in terms of lung injury scores, namely, the PaO2/FiO2 ratio, immune profiles, and serum inflammatory markers and cytokines in severe ARDS caused by COVID-19 pneumonia.

## Methods

### Subjects and Methods

This was an investigator-initiated academic compassionate trial conducted at the Taipei Medical University Hospital, Taipei, Taiwan.

### Patient Enrollment

Patients who met the following criteria were recruited: 1) age 18–90 years old; 2) laboratory confirmed COVID-19 infection by using a reverse transcriptase polymerase chain reaction (RT-PCR) assay from nasopharyngeal specimen; 3) severe COVID-19 pneumonia who meet Berlin’s criteria of severe ARDS and/or shock requiring inotropes; and 4) persistent or deteriorating lung injury with hyper-inflammatory states despite use of dexamethasone and anti-IL-6 antibody, tocilizumab. The patients who were recruited in the study were not vaccinated at that time and were treated according to the local clinical practice protocol, which included 6 mg/day of dexamethasone for those classified as having severe illness and an additional 8 mg/kg of tocilizumab for steroid-refractory patients. Patients who were allergic to dimethyl sulfoxide (DMSO; a component of pcMSCs), recipients of previous stem cell therapy, or those with pre-existing terminal illness, in need of extracorporeal membrane oxygenation (ECMO), dialysis dependent, or with multiorgan failure status were excluded. According to local government law and legislation, five subjects were allowed to receive compassionate treatment with pcMSC. Nineteen compatible subjects hospitalized in the ICU wards for COVID-19 and severe lung injury with a Murray’s lung injury score of >1.7 were assigned to the control group.

### Cell Preparation and Treatment Protocol

The detailed procedures for the preparation of the pcMSC are listed in the **Supplementary File**. Subjects received two intravenous infusions of 200 ± 20 × 10^5^ pc-MSCs on Days 0 and 4. The patients were premedicated with antihistamines before each cell treatment. The clinical status of the patients was closely monitored after cell treatment and in serial follow-up for 2 months after therapy. The best standard of care was provided in both groups, following the current institutional COVID-19 guidelines.

### Analysis of Viral Load by SARS-CoV-2 RT-PCR

The RealStar SARS-CoV-2 RT-PCR kit (Altona Diagnostics GmbH, Hamburg, Germany) was used to detect the SARS-CoV-2-specific E gene. The assay was performed following the instructions of the manufacturer, using nasopharyngeal swab samples collected from enrolled subjects on Day 0 and every 6 days until the cycle threshold (ct) >30 was reached or a negative PCR was detected.

### Analysis of Inflammatory Cytokines, Chemokines, and Immune Profiles in Peripheral Blood

The multiplex cytokine assay (Aimplex Biosciences, CA) was performed according to the instructions of the manufacturer to determine the levels of 18 inflammation-related proteins in serum of subjects treated with pc-MSC on Days 0, 4, and 8. Approximately 45 μl of serum from the pc-MSC treated subjects on Days 0, 4, and 8 was added to 96-well plates with beads labeled with specific antibodies for 18 inflammation-related proteins, namely, Interleukin (IL)-1β, IFN-γ, TNF-α, IL-2, IL-6, IL-10, IL-13, IL-5, IL-4, IL-17A, IL-18, and IL-22. After incubation for 1 h, the bead complexes were washed and then a biotinylated detection antibody was added for 30 min, which was followed by avidin-PE. After washing, the complexes were analyzed by multiple parameter cytometry (ID7000, Sony Biotechnology, WA, USA).

Immune profiles were obtained from multiple parameter cytometry (ID7000) to determine the proportions of T cells, B cells, NK cells, monocytes, dendritic cells, and their subsets in the peripheral blood of subjects treated with pc-MSC on Days 0, 4, and 8 (**Supplementary Figure 1**). In summary, peripheral blood mononuclear cells (PBMC) were isolated from subjects treated with pc-MSC by density gradient centrifugation and stained with a specific antibody labeled with a fluorescent dye; the designed panel is shown in **Supplementary Table 1**. After washing, the MFI of the sample was analyzed using the Sony ID7000.

### Statistical Methods

Statistical analysis was conducted using Prism 9.0 (Graphpad Software, CA, USA). To examine whether the data were normally distributed, the Kolmogorov–Smirnov test was used first. Comparisons of adverse effects, demographics, clinical characteristics, comorbidities, and concomitant treatments between the two groups were made using Fisher’s exact test and Wilcoxon’s two-sample tests for categorical and continuous variables, respectively. The Wilcoxon signed-rank test or Mann–Whitney test was used to compare the continuous variables of the paired or unpaired groups that were non-normally distributed, respectively. Survival and survival in the absence of serious adverse events (SAE) (SAE-free survival) were estimated in each group with Kaplan–Meier survival estimates. Log-rank tests were used to compare hazard ratios between groups.

## Results

### Clinical Characteristics and Clinical Courses

**Figure 1:**
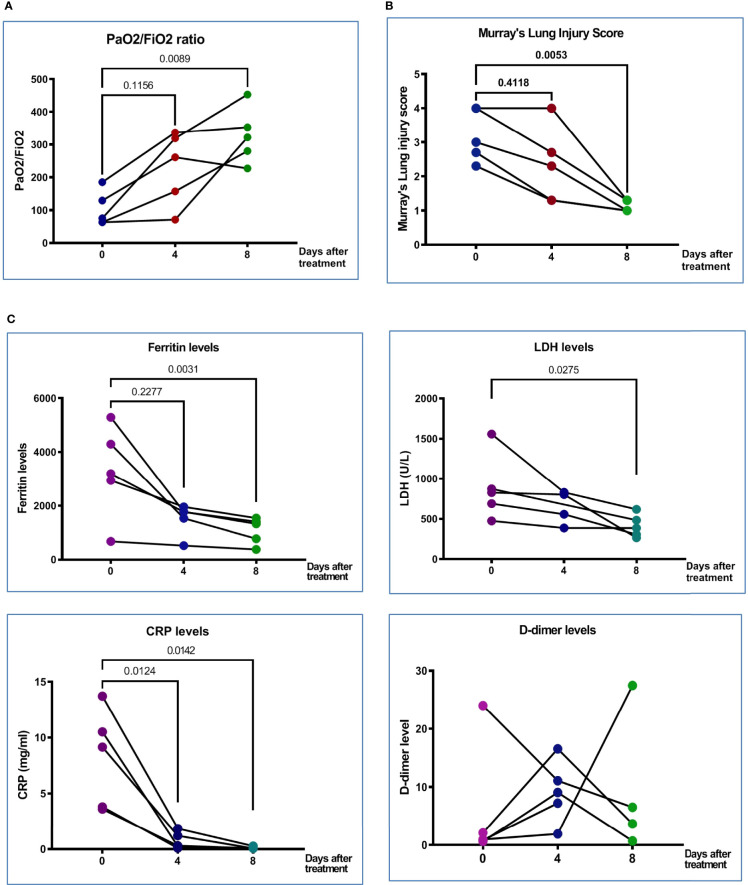
Clinical characteristics and clinical outcomes of patient with COVID-19. **(A)** PaO_2_/FiO_2_ ratio at Day 0 (before treatment), Day 4 (after the first treatment with MSC), and Day 8 (after second treatment with MSC) in five treated patients. **(B)** Murray’s lung injury score was shown before and after treatment; timing of after was adjusted as one week after recruitment. **(C)** Serum proinflammatory markers, derritin, LDH, CRP, and D-dimer on Days 0, 4, and 8 after pcMSCs therapy of five treated patients. The P value was as indicated. Nonparametric Wilcoxon sign-rank test.

**Figure 2:**
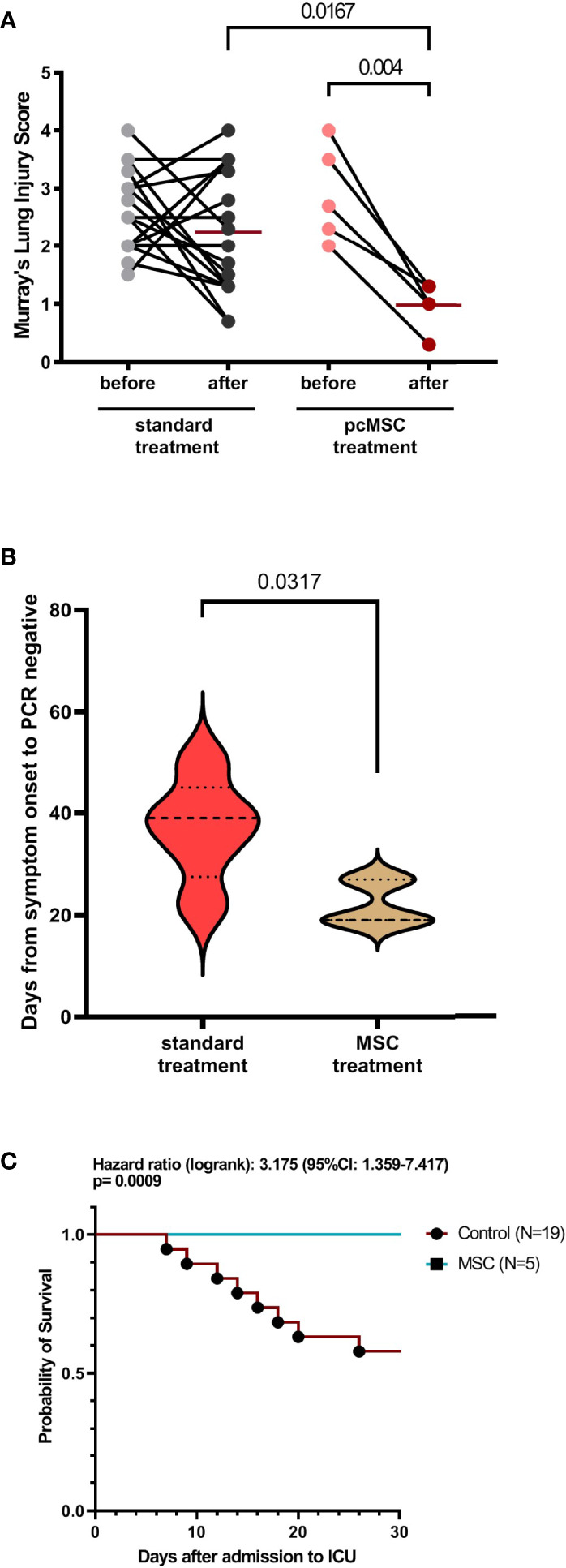
Clinical characteristics of COVID-19 patient with standard or MSC treatment. **(A)** Murray’s lung injury score of standard treatment and MSC treatment group was shown as before and after treatment; timing of after was adjusted as one week after recruitment. **(B)** Duration of viral clearance by detecting viral COVID molecular testing by PCR in control and MSC treated group. **(C)** Survival analysis of control and MSC treated group.

From 14 May to 18 June 2021, subjects with severe refractory deteriorating COVID-19 pneumonia (n = 5), despite standard treatments, consented to receive compassionate pc-MSC treatment. Twenty-four subjects were hospitalized in the ICU for COVID-19-related pneumonia during this period. The demographics and baseline clinical conditions of these patients are presented in **Table 1**. The predominance of male sex with younger age and a higher body mass index (BMI) was observed in the pc-MSC group. There were no significant differences in concomitant treatments, white blood cell count, lymphocyte count, and inflammatory markers between the two groups (**Table 2**). Three subjects (60%) in the pc-MSC group and 11 subjects (57.9%) in the control group received invasive mechanical ventilation, and 2 subjects (40%) in the MSC group and 8 (42.1%) in the control group received high flow oxygen therapy (FiO2 >60%) *via* a high flow nasal cannula before starting treatment. A total of 9 deaths occurred in the control group on Day 28 after enrollment. There were no significant differences between the control and the pc-MSC group (MSC) in terms of the number of comorbidities, baseline hypoxemia, Murray lung injury scores, PaO2/FiO2 ratio, or length of stay in the ICU. Treatment with pc-MSC treatment achieved clinical improvement in terms of the PaO2/FiO2 ratio (**Figure 1A**) and Murray lung injury score (**Figure 1B**) with concomitant decreases in systemic hyper-inflammatory states, in terms of serum levels of ferritin, CRP, and LDH (**Figure 1C**) within 7 days of therapy. There were no significant changes in serum D-dimer levels with pc-MSC treatment (**Figure 1C**). In contrast, 9 patients in the control group presented with progressive deterioration of the Murray lung injury score (**Figure 2A**) within 7 days of therapy. Treatment with pc-MSC also enhanced viral clearance (**Figure 2B**) and sustained survival compared to the control group (**Figure 2C**).

**Table 1:** Demographics and baseline clinical conditions of the treated patients.

	Patient 1	Patient 2	Patient 3	Patient 4	Patient 5
Age	66	78	41	46	46
Gender	F	F	M	M	M
Weight (kg)	63	70.6	83	104	114
BMI (kg/m^2^)	27.45	27.24	29.41	36.85	39.45
Smoking	NIL	NIL	NIL	6 pack-year	NIL
Comorbidities	Hypertension Dyslipidemia	Hypertension DM Breast cancer (post operation) Colon cancer (post operation)	NIL	Hypertension DM CABG	Hypertension DM
Clinical presentation	Fever, Dry cough	Cough	Fever, Cough	Fever	Cough, Breathlessness
Days from symptoms onset to admission	2	1	2	13	7
Days from symptoms onset to ICU admission	8	1	18	19	8
Days from admission to critical care transfer	6	1	16	6	1
Days from admission to intubation	11	2	HFOT	HFOT	1
Duration of MV used	10	8	HFOT	HFOT	16
Days of MV used after first infusion	10	8	HFOT	HFOT	13
SOFA score prior to infusion	4	8	4	4	5

F, female; M, male; DM, Diabetes Mellitus; CABG, Coronary Arterial Bypass Graft; MV: mechanical ventilation; HFOT, High flow oxygen therapy; BMI, Body mass index; ICU, intensive care unit.

**Table 2:** Demographics and baseline characteristics of the pc-MSC treated and standard treatment group with serum inflammatory markers and clinical outcome.

Characteristics and treatments		pc-MSC treatment (n = 5)	Standard treatment (n = 19)	p-value
Age, years, mean (SD)		55.4 ± 7.1	70.5 ± 2.2	0.012
Gender				0.24
	Male	3 (60%)	16 (84.2%)	
	Female	2 (40%)	3 (15.7%)	
PaO2/FiO2 ratio at enrollment, mean (IQR)		108.7 ± 13.6	148.5 ± 15.3	0.16
Murray’s lung injury score, n (%)				0.6
	Moderate-to-severe >1 <2	0 (0%)	1 (5.2%)	
	Severe>2	5 (100%)	18 (94.73%)	
BMI, kg/m^2^, mean (SD)		32.0 ± 2.5	25.8 ± 1.4	0.04
Smoker (including former smoker), n (%)		0	4 (21.0%)	0.54
Comorbidities, n (%)		4 (80%)	13 (68.4%)	0.93
	DM	3 (60%)	5 (26.3%)	0.28
	Hypertension	4 (80%)	7 (36.8%)	0.14
	Obesity (BMI >30)	2 (40%)	5 (26.3%)	0.6
	Cancer	1 (20%)	1 (5.2%)	0.38
	Heart disease	1 (20%)	4 (21.0%)	0.99
Concomitant treatments, n (%)		5 (100%)	19(100%)	0.99
	Heparin, Therapeutic dose	5 (100%)	16 (84.2%)	0.99
	Remdesivir	5 (100%)	3 (15.8%)	0.001
	Corticosteriods	5 (100%)	19 (100%)	0.99
	Tocilizumab	5 (100%)	19 (100%)	0.99
Intubation at enrollment		3 (60%)	11 (57.9)	0.99
Mortality, n (%)		0	8 (42.1%)	0.13
At the time of enrollment				
WBC (×1,000/μl)		12.25 ± 3.22	7.71 ± 0.89	0.065
Lymphocytes (×1,000/μl)		8.84 ± 3.46	11.92 ± 2.12	0.501
CRP (mg/dl)		6.6 ± 2.74	8.17 ± 1.39	0.613
Ferritin (ng/ml)		2345 ± 441.9	2423 ± 449	0.933
D-Dimer (ug/ml)		5.77 ± 4.58	3.61 ± 1.47	0.561
Procalcitonin (ng/ml)		0.39 ± 0.27	0.57 ± 0.26	0.746
LDH (U/L)		679.2 ± 84.06	569.9 ± 37.41	0.209

WBC, white blood cells; LDH, Lactate Dehydrogenase; CRP, C-reactive protein.

### Analysis of Immune Profiles, Inflammatory Cytokines, and Chemokines Levels in Peripheral Blood

**Figure 3:**
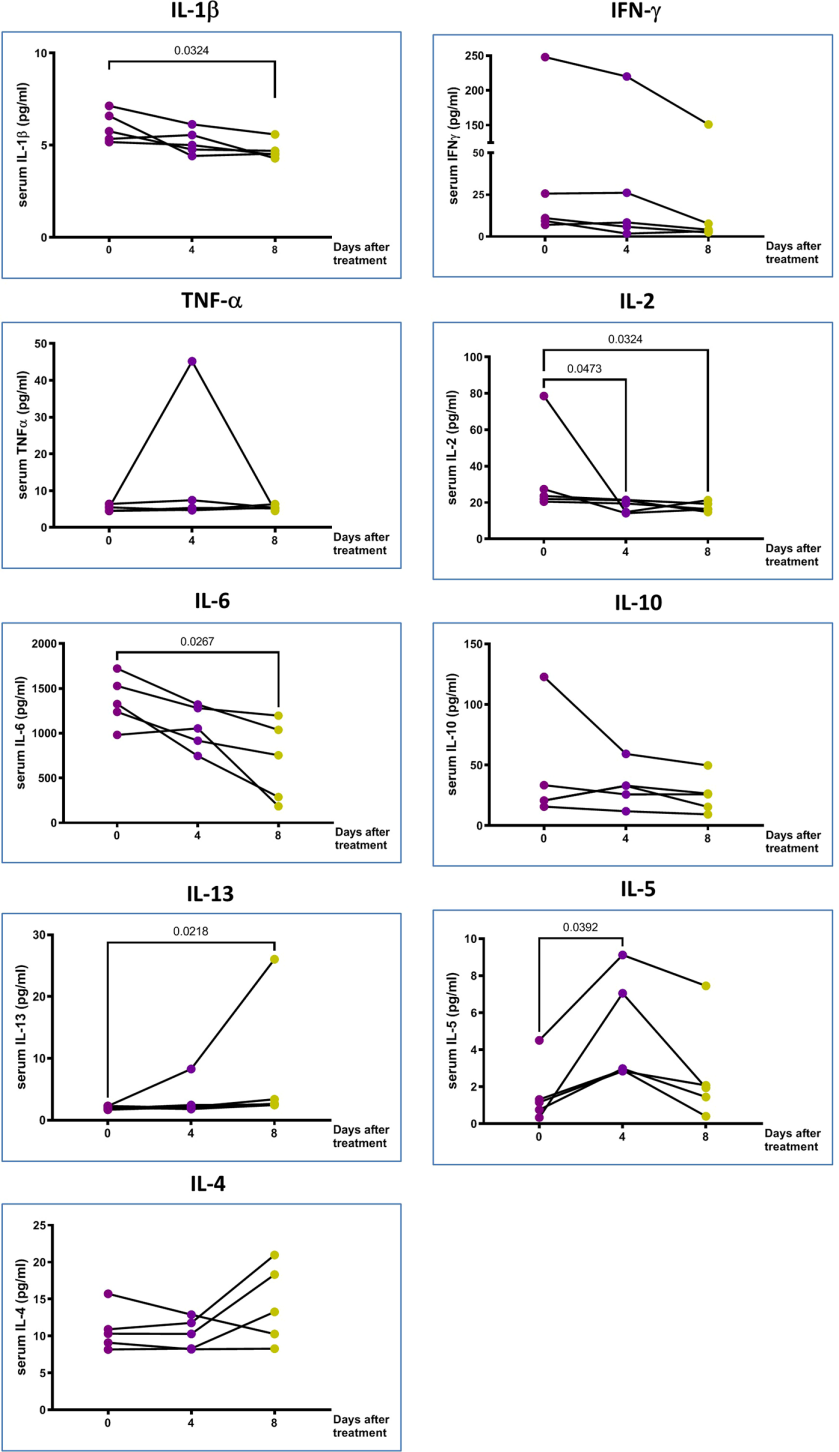
The proinflammatory markers in the peripheral blood. IL-10 and type 2 cytokines changes of 5 treated patients in Days 0, 4 and 8 after pcMSCs therapy. The P value was as indicated. Nonparametric Wilcoxon sign-rank test.

The multiplex cytokine assay showed that the plasma levels of IL-6 and IL-2 significantly decreased on Day 4, and IL-1β and IFN-γ decreased significantly on Day 8 of pc-MSCs treatment (**Figure 3**). There was no significant change in plasma levels of IL-17A (not shown), TNFα, IL-18 (not shown), or IL-22 (not shown). There was also a significant increase in type 2 cytokines, IL-5 and IL-13, after pc-MSC treatment (**Figure 3**). IL-10, a known inhibitory cytokine, was shown to have a trend of decreasing after pc-MSC treatment.

Peripheral blood samples from patients with the pc-MSC treatment group were analyzed by high-dimensional flow cytometry and the immune cell cluster was visualized using t-Distributed Stochastic Neighbor Embedding (t-SNE) (**Supplementary Figure 2A**). Its distribution was also checked after treatment and showed a significant increase in the proportions of Treg and B cells with a concomitant reduction in the proportions of plasma/plasmablast cells and monocytes (**Supplementary Figure 2B**).

**Figure 4:**
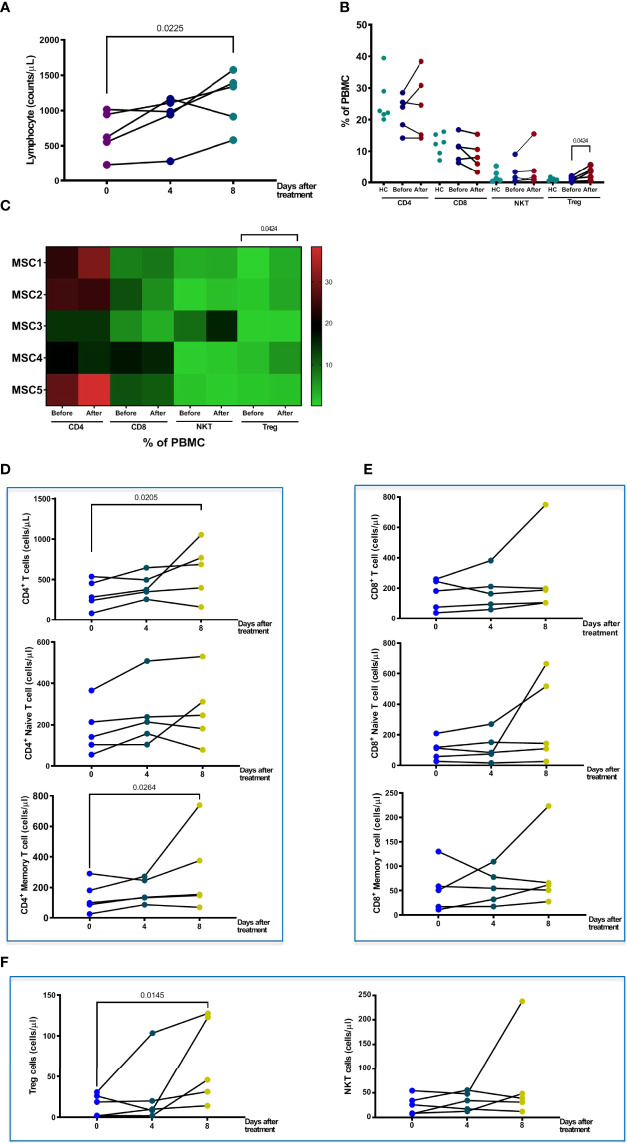
Changes in T cells and sub-populations after pcMSC treatment. **(A)** Total lymphocyte count changes after Days 0, 4, and 8 of treatment. **(B)** The proportions of CD4, CD8, NKT, and Treg cells before and after treatment, compared with normal healthy individuals. **(C)** Distribution of T cells subpopulations was shown in the heat map. **(D)** The absolute cell number of total CD4^+^ T cell, naïve CD4^+^ T cell, and CD4^+^ memory cell increased after treatment. **(E)** No change in absolute cell number of CD8^+^ T cell, CD8^+^ Naïve T cell, or CD8^+^ memory T cell. **(F)** The absolute cell number of Treg cells was significantly increased after Day 8 of treatment. The P value was as indicated. Nonparametric Wilcoxon sign-rank test.

Patients in the pc-MSC treatment group had lower absolute lymphocyte counts in the peripheral blood (667.1 + 137.1/μl, n = 5) compared with healthy controls (1,798.6 + 145.2/μl, n = 5, P <0.01). Absolute lymphocyte counts increased after pc-MSC treatment (1,161 + 180.7/μl, P <0.05, n = 5) (**Figure 4A**). There were no significant changes in the proportions of CD4^+^ T cells, CD8^+^ T cells, or natural killer (NKT) cells compared with healthy controls after pcMSC therapy (**Figure 4B**). A heat map was created to show the changes in the proportion of the T cell subpopulation before and after treatment. Patients initially had lower counts of CD4^+^ T and CD8^+^ T cells (321.7 ± 80.1/μl, and 160.4 ± 44.5/μl, respectively, n = 5) compared with healthy controls (813.5 ± 76.5/μl and 296.5 ± 53.1/μl, respectively, n = 5, P <0.05) (**Figure 4C**). Treatment with pc-MSC significantly increased the absolute cell number of total CD4^+^ T cells (655.5 ± 163.6/μl, n = 5, P <0.05) and the subpopulations of CD4^+^ naïve T cells and CD4^+^ memory T cells (**Figure 4C**), but not absolute cell number of total CD8^+^ T cells (269.2 ± 121.7/μl, n = 5) or its subpopulations (**Figure 4D**). Treg cells proportions (**Figure 4B**) and absolute counts (**Figure 4E**) increased significantly. NKT cells did not change significantly with pc-MSC treatment (**Figure 4E**).

**Figure 5:**
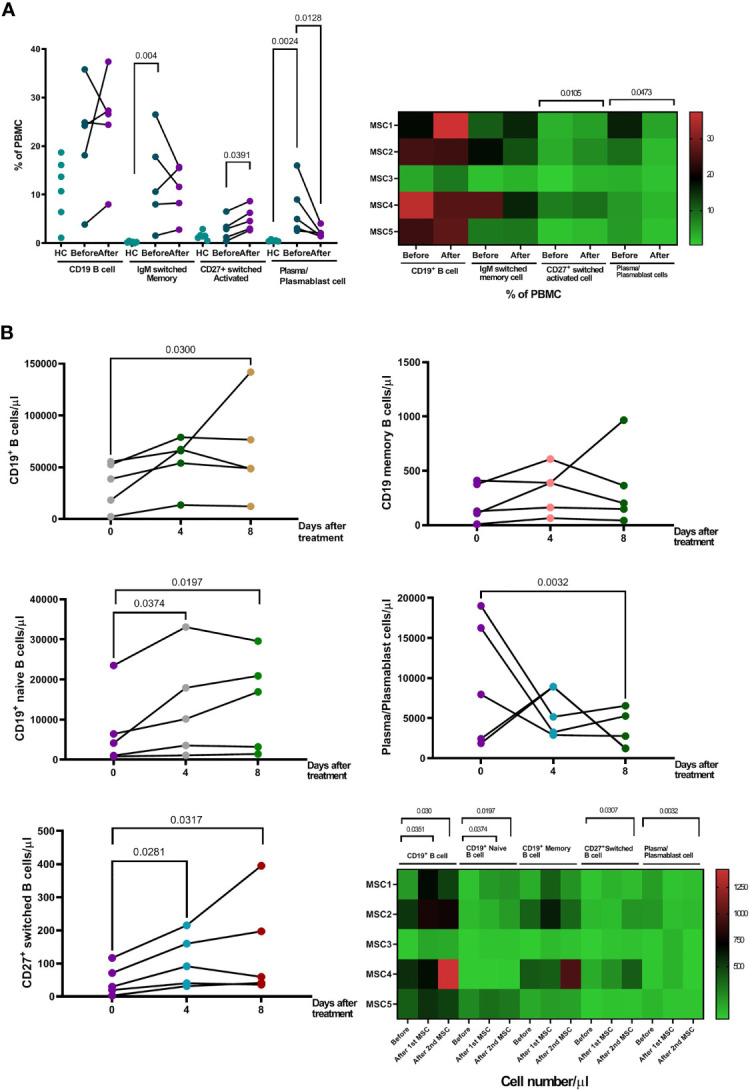
Changes of B cell populations after pcMSC treatment. **(A)** High proportion of IgM^+^ switched memory B cells and CD27^+^CD38^+^ plasma/plasmablast cells in the peripheral blood, compared to healthy controls, before and after treatment. Treatment with pcMSCs increased the proportions of CD27^+^ switched activated B cell and decreased proportion of plasma/plasmablast cells. **(B)** The absolute number of B cells, CD19^+^ total B cell count, CD19^+^ Naïve B cells count, and CD27^+^ switched B cell were significantly increased after D4 and D8 of treatment. The absolute number of Plasma/Plasmablast cell was significantly reduced after Day 8 of treatment. The P value was as indicated. Nonparametric Wilcoxon sign-rank test.

Patients initially had a higher proportion of IgM^+^ memory B cells and CD27^+^CD38^+^ plasma/plasmablast cells in their peripheral blood, compared to healthy controls (**Figure 5A**). Treatment with pcMSC treatment significantly suppressed the proportion and absolute number of plasma/plasmablast cells with CD27^+^CD38^+^ (**Figures 5A, B**), and significantly increased the absolute number of CD19^+^ B cells, CD19^+^ naïve B cells, and increased the proportion and the absolute number of activated B cells switched to CD27+ (**Figure 5B**). Treatment with pcMSC treatment did not change the proportion of IgM^+^ memory B cells.

**Figure 6:**
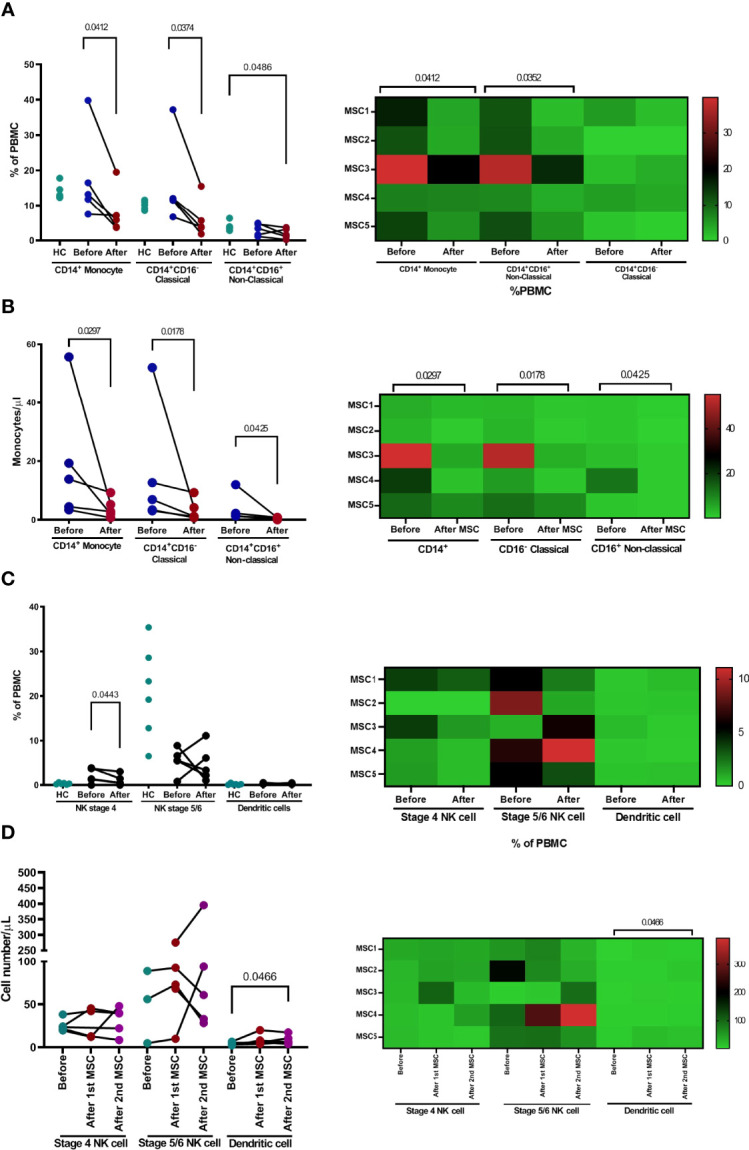
The changes of monocyte and NK cell population after pcMSC treatment. **(A)** The proportion and **(B)** the absolute number of total monocytes, classical monocytes, and non-classical monocytes were reduced after treatment. **(C)** The proportions of dendritic cells, NKT cells, and subpopulations were not significantly changed after treatment, compared with corresponding healthy controls. **(D)** The absolute number of dendritic cells, but not NK cells, or subpopulations, increased after Day 8 of treatment. *P <0.05 and **P <0.01, nonparametric Wilcoxon sign-rank test.

The proportions of CD14^+^, CD14^+^CD16^+^, or CD14^+^CD16^−^ monocyte subpopulations were not significantly different between patients and healthy controls (**Figure 6A**). Treatment with pcMSC significantly decreased the proportion of CD14^+^ and CD14^+^CD16^−^ subpopulations and achieved a significantly lower proportion of CD14^+^CD16^+^ subpopulations compared with healthy controls (**Figure 6A**). The absolute number of CD14^+^ monocytes and their subpopulations, CD14^+^CD16^−^ and CD14^+^CD16^+^ were significantly decreased after pcMSC treatment (**Figure 6B**).

There was no significant change in the proportion of stage 4, stage 5–6 NK cells, or dendritic cells (**Figure 6C**). pcMSC treatment only significantly increased the absolute number of dendritic cells (**Figure 6D**).

### Adverse Events

No SAEs were observed in the pc-MSC group after an 8-week follow-up.

## Discussion

In this study, we showed that pcMSC treatment was safe and effective in patients with severe COVID-19 who developed ARDS. Nine of the 19 patients in the control group died by day 30, indicating a 47% mortality at our institution, which was consistent with the reported mortality rate of ~50% of critically ill COVID-19 patients (10), while none of the patients treated with pc-MSC died on day 28. It is interesting to note that 2 of the 5-treated patients did not require mechanical ventilation. These patients had identified risk factors for respiratory failure in severe COVID-19, such as higher BMI and a poor PaO2/FiO2 ratio.

All patients had elevated blood inflammatory markers, namely, CRP, D-dimer, LDH, and ferritin, as previously reported (26, 27), although the patients had received dexamethasone and tocilizumab as anti-inflammatory treatments. Treatment with pc-MSC suppressed the hyper-inflammatory state with markedly reduced ferritin, LDH, and CRP levels after 7 days.

SARS-CoV-2 RNAs act as pathogen-associated molecular patterns, detecting toll-like receptors, triggering downstream cascades in innate immune cells, resulting in the production of pro-inflammatory cytokines, such as IL-1β, IL-6, and IFN-γ that induce the synthesis of several liver defense proteins, namely, CRP, LDH, and ferritin (28). These multifunctional peptides, especially LDH and ferritin levels, are independent factors in predicting disease severity and mortality (29, 30). Ferritin, through NF-κB (31) creates a vicious loop by further increasing the production of pro-inflammatory cytokines, IL-1β, IL-6, and IFN-γ, contributing to the development of a cytokine storm syndrome (CSS). The reduction in serum ferritin levels after pcMSC treatment was associated with a reduction in IL-1β, IL-6, and IFN-γ serum levels, suggesting that pcMSC effectively broke the vicious cycle and attenuated the cytokine storm syndrome, resulting in a significant attenuation of the severity of COVID-19 pneumonia in terms of Murray’s lung injury scores.

MSC therapy offers a promising treatment option for severe lung diseases caused by autoimmune, sepsis, and COVID-19 infection (18–20). The underlying cellular and molecular mechanisms of MSC-mediated immunomodulation, although still elusive, are proposed to act *via* a synergy of cell contact-dependent mechanisms and soluble factors (13, 32), *via* cytokine-dependent and cytokine-independent functional changes of monocytes/macrophages, dendritic cells, T cells, B cells, and NKT cells (14–16, 33). Peripheral blood immune profiles with tSNE cell clusters revealed a significant change in monocytes, Th cells, B cells, Treg cells, and plasma/plasmablast cell subpopulations after pc-MSC treatment.

Monocytes and macrophages, being the most important innate immune cells, respond mainly to SARS-CoV-2 infections by producing pro-inflammatory mediators through ACE2-independent and ACE2-dependent pathways to remove pathogens and repair tissue injury. However, dysregulation of their functions can cause acute respiratory distress syndrome and cause damage to other vital organs, including the heart (34). During SARS-CoV-2 infection, the subpopulation of circulating CD14^+^CD16^+^ monocytes exhibits a notable contribution to the development of cytokine storms, orchestrating a high level of TNF-α, IL-10, and IL-6 production, which is ultimately related to clinical deterioration and thus, increases the likelihood of admission to the ICU (2, 35). The decrease in the absolute number of subpopulations CD14^+^ CD16^+^ may be attributed to the attenuation of hyper-inflammation responses in the pcMSC treatment group.

In severe COVID-19, lymphopenia (absolute counts <1,000 cells/μl) is considered an indicator of disease severity (36), therapeutic response (37), and disease outcome (38). High levels of pro-inflammatory cytokines such as TNF-α and IL-6 could induce lymphocyte deficiency (38). Our patients had absolute lymphocyte counts <1,000/μl before treatment. These measured lymphocyte numbers in the peripheral blood increased significantly after treatment, probably resulting from the inhibitory effect of pcMSC on pro-inflammatory cytokines such as IL-6.

Lymphocytes in the peripheral blood are critical to generating early control, viral clearance, and disease resolution after SARS-CoV-2 infection (39, 40). Regulatory T cells (Treg) play an important role in the prevention of excessive immune responses to SARS-CoV-2 infection (41). On analysis of the T lymphocyte subpopulations, our patients had initially lower counts of CD4^+^ and CD8^+^ T cells, but not in the proportion of CD4^+^, CD8^+^ NKT, or Treg cells, compared with age-matched healthy controls. Treatment with pcMSC significantly increased the proportion and absolute number of CD4^+^ T and Treg cells. Among CD4^+^ T cell subpopulations, memory T cells are crucial in viral clearance during events associated with re-infection. Although memory T cells were not identified as SARS-CoV2-specific, the presence of more memory T cells may suggest that adaptive immunity is being developed. MSCs facilitate Treg cell formation (14, 42) by inducing the formation of Tregs from conventional T cells (43, 44). The increase in Treg cells is considered responsible for the suppression of hyperinflammatory responses mediated by pcMSC in our patients. Furthermore, MSC treatment induces a switch from pro-inflammatory Th1 cells to anti-inflammatory Th2 cells (45). Increased serum levels of IL-5, IL-4, and IL-13 after pcMSC treatment may support this notion.

An effective immune response to the SARS-CoV infection depends on the activation of CD8^+^ cytotoxic T cells through the killing of virus-infected cells (46). However, although there was a trend for CD8^+^ T cells to increase with pcMSC treatment, statistical significance was not reached. Our data on the role of NK cells were limited. In one study, there were no differences in NK cell levels between responders and nonresponders before and after treatment (47). In another study, NKT cell levels showed an increasing trend among survivors, while in non-survivors, reductions were observed (48). In our study, there was no significant change in NK cells after pcMSC treatment.

B cell-mediated antibody responses through the coordination of other adaptive immune responses, namely, CD4^+^ and CD8^+^ T cells, are essential for effective control of COVID-19 infection (49). B cells contribute to the antiviral immune response by first rapidly releasing germline or near-germline antibodies from plasmablasts *via* an extrafollicular pathway. In patients with COVID-19 infection, there are increased portions of IgM^+^ memory B cells and CD27^+^CD38^+^ plasma/plasmablast cells circulate in the peripheral blood, compared to healthy controls, suggesting a protective memory response occurred (5). Upon appropriate stimulation of cytokines, B cells undergo class switching and/or enter germinal centers within secondary lymphoid organs to undergo affinity maturation. This maturation process produces long-lived plasma and memory B cells capable of responding to secondary challenges with homotypic or heterotypic antigenic challenges. pcMSC treatment significantly increased the proportion and absolute number of CD27^+^-switched activated B cells and concomitantly suppressed the proportion and absolute number of CD27^+^CD38^+^ plasma/plasmablast cells. These results suggest that pc-MSC treatment induced a shift of B cells from a protective memory state to an active response to COVID-19 infection by enhancing the generation of virus-specific immunoglobulin switched B cells or long-lived plasma cells (50).

### Study Limitations

The study was a small sample-sized study without a true randomized control group to provide strong evidence of treatment benefit. According to local government laws and legislation, compassionate research for stem cells is limited to 3 patients. Due to the pandemic, our study was allowed to enroll 5 patients. At the time of treatment, all patients had already been treated with antiviral and anti-inflammatory agents, including dexamethasone and tocilizumab. Thus, it was difficult to elucidate immune modulatory effects purely due to pcMSC. Despite these limitations, our treatment showed a desirably favorable prognosis, biochemical responses, and immune profiles with pcMSCs therapy in the treated patients.

## Data Availability Statement

The original contributions presented in the study are included in the article/**Supplementary Material**. Further inquiries can be directed to the corresponding authors.

## Ethics Statement

The studies involving human participants were reviewed and approved by the Taipei Medical University Institutional Review Board. The patients/participants provided their written informed consent to participate in this study.

## Author Contributions

M-CC and KL wrote the manuscript. H-PK, Y-HH, C-MW and C-LC contributed to the design and implementation of the research. K-LC, ST, P-LW involved in clinical care and collecting clinical specimen. Y-RL, WC, Y-HH and M-JL carried out laboratory analysis. H-PK, C-LC and C-MW revised the manuscript and provided additional comments. All authors read and approved the final manuscript.

## Funding

This study was supported by the Taiwan Ministry of Science and Technology grants, 109-2314-B-038-097 and 108-2314-B-038-134-MY2.

## Conflict of Interest

The authors declare that the research was conducted in the absence of any commercial or financial relationships that could be construed as a potential conflict of interest.

## Publisher’s Note

All claims expressed in this article are solely those of the authors and do not necessarily represent those of their affiliated organizations, or those of the publisher, the editors and the reviewers. Any product that may be evaluated in this article, or claim that may be made by its manufacturer, is not guaranteed or endorsed by the publisher.

## Abbreviations

ARDS, severe acute respiratory distress syndrome; pcMSC, placenta-derived mesenchymal stem cell; LDH, lactate dehydrogenases; CRP, C-reactive protein; DMSO, dimethyl sulfoxide; ECMO, extracorporeal membrane oxygenation; ICU, intensive care unit; PCR, polymerase chain reaction; t-SNE, t-Distributed Stochastic Neighbor Embedding.
