# Supplementary material for: pcMSC Modulates Immune Dysregulation in Patients With COVID-19-Induced Refractory Acute Lung Injury
Source: Front Immunol. 2022 Apr 29;13:871828. doi: 10.3389/fimmu.2022.871828 (PMC9109702; doi:10.3389/fimmu.2022.871828)
Supplement: Supplementary file 1 [file DataSheet_1.docx]

Supplementary Material

**Appendix-S1: METHODS**

**Cell preparation**

An average of MatriPlax were administered per infusion. The viability of pc-MSCs at the time of product release for administration was found to be 88±2% by trypan blue and 90 %±2% by flow cytometry using fixable viability stain. No differences in cell dose, cell viability, or degree of apoptosis were observed between pc-MSCs prepared for the first or second infusion. Stability studies demonstrated stability of the pc-MSC investigational product for up to 8 hours after thawing and preparation, as assessed by cell count, viability by trypan blue and flow cytometry, and apoptosis assessed by flow cytometry. Cell surface marker analysis demonstrated a typical surface marker profile characteristic of MSCs: CD90 of 99% ±0.2%, CD73 of 95%±0.2%, and CD34/CD45 of 0%±0.1%.

**Appendix-S2:**

**
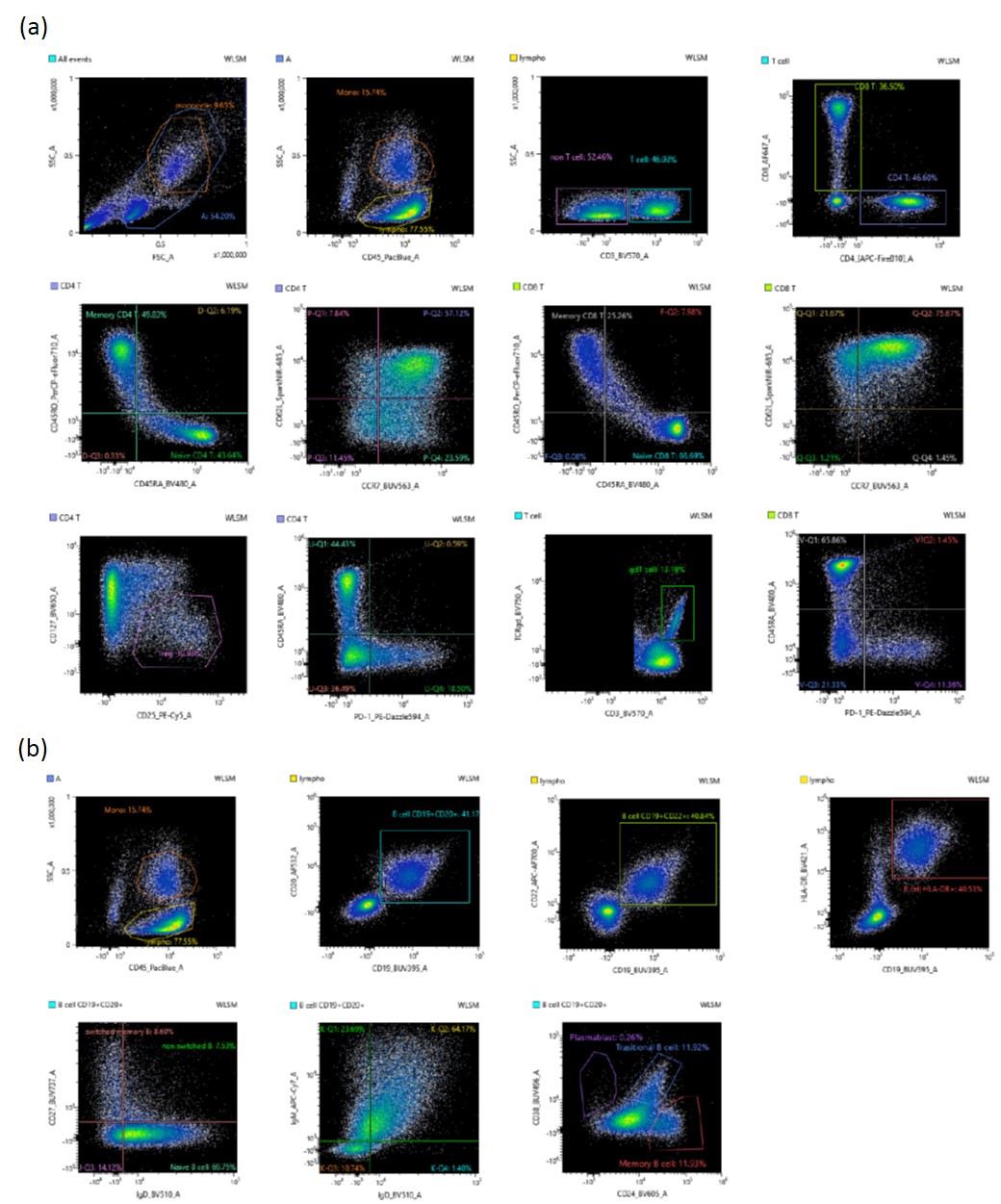
**

**
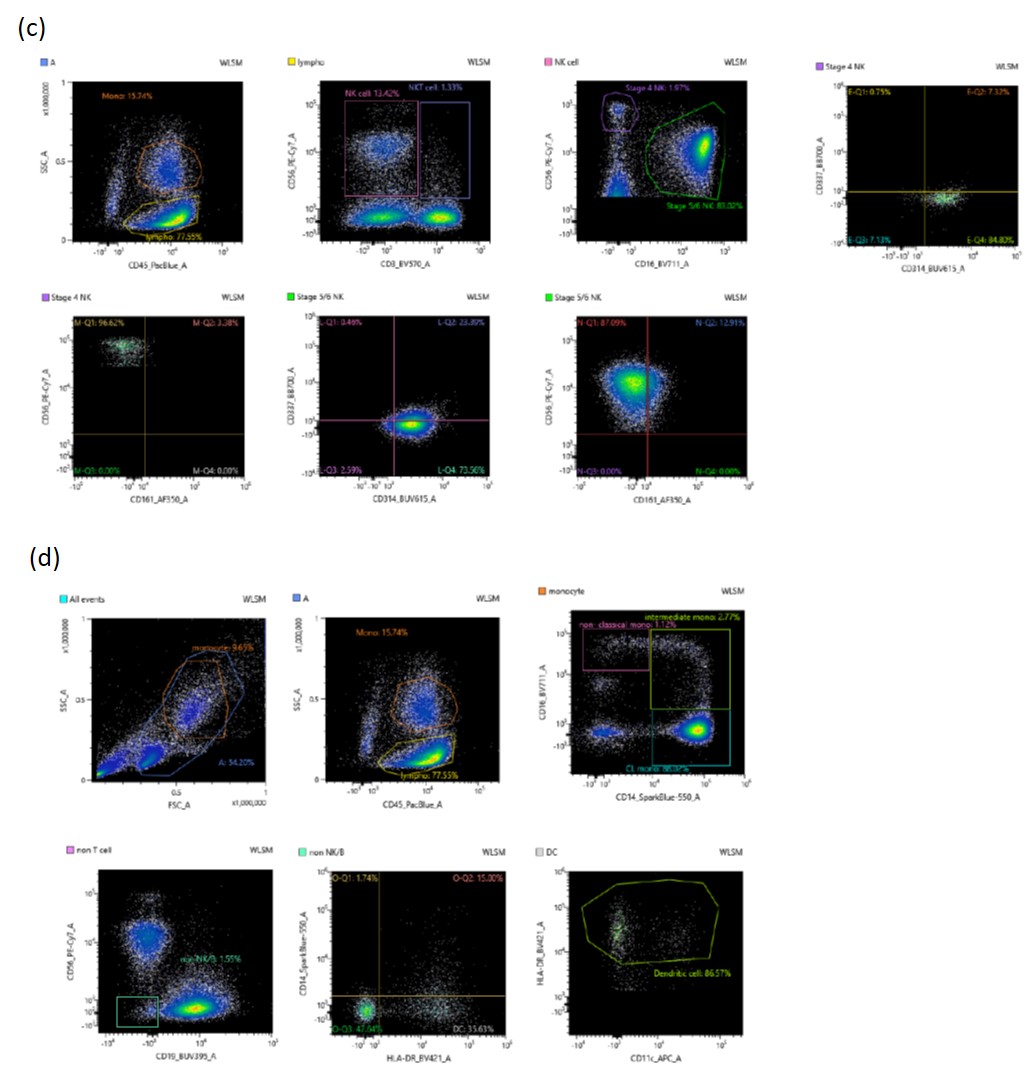
Figure S1 Gating strategy of immune cells** (a) T cell (b) B cell (c) NK population (d) Monocyte population

**
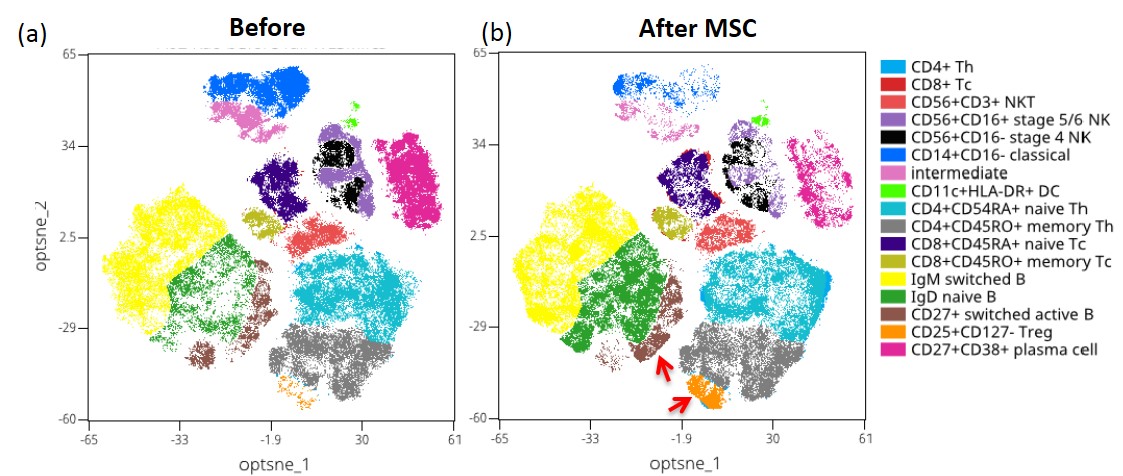
Figure S2**

**The cell clusters of the peripheral blood mononuclear cells of patients shown by t-SNE.** (a) the proportion of immune cells before treatment with pc-MSCs. (b) the changes in cell populations of each cell cluster after treatment: increased in Treg cell and B cell sub-clusters with a concomitant reduction in sub-clusters of plasma/plasmablast cell and monocyte.
